# Recent Advances in Continuous-Flow Particle Manipulations Using Magnetic Fluids

**DOI:** 10.3390/mi10110744

**Published:** 2019-10-31

**Authors:** Xiangchun Xuan

**Affiliations:** Department of Mechanical Engineering, Clemson University, Clemson, SC 29634-0921, USA; xcxuan@clemson.edu; Tel.: +1-864-656-5630

**Keywords:** negative magnetophoresis, paramagnetic solution, ferrofluid, particle deflection, particle focusing, particle enrichment, particle separation, particle medium exchange

## Abstract

Magnetic field-induced particle manipulation is simple and economic as compared to other techniques (e.g., electric, acoustic, and optical) for lab-on-a-chip applications. However, traditional magnetic controls require the particles to be manipulated being magnetizable, which renders it necessary to magnetically label particles that are almost exclusively diamagnetic in nature. In the past decade, magnetic fluids including paramagnetic solutions and ferrofluids have been increasingly used in microfluidic devices to implement label-free manipulations of various types of particles (both synthetic and biological). We review herein the recent advances in this field with focus upon the continuous-flow particle manipulations. Specifically, we review the reported studies on the negative magnetophoresis-induced deflection, focusing, enrichment, separation, and medium exchange of diamagnetic particles in the continuous flow of magnetic fluids through microchannels.

## 1. Introduction

Lab-on-a-chip devices have been proven to be very useful in handling various types of samples for a wide range of chemical, environmental, and biomedical applications [1,2,3,4]. In these microfluidic systems, many different forces have been demonstrated to control the motion of particles (a general term for any micro/nano-sized small objects that may cover polystyrene (PS) beads, water- or oil-based droplets, bacteria, fungi, mammalian cells, etc.), among which electric [5], magnetic [6], acoustic [7], and optical [8] fields are the most often used. While every force field has its own advantages, magnetic manipulation of particles is the simplest and cheapest [9,10,11]. Moreover, the use of permanent magnets eliminates the issue of fluid heating that accompanies nearly every other field-induced technique. It is therefore potentially well suited to handle bioparticles [12,13,14]. However, traditional magnetic techniques require the particles to be manipulated being magnetizable [15,16,17], which renders the magnetic labeling necessary, since majority of the particles (except red blood cells [18] and magnetotactic bacteria [19]) are diamagnetic in nature. This labeling step not only extends the sample pre- and post-processing times but also increases the operational cost. Recently, there has been a significantly growing interest in the use of magnetic fluids for the label-free manipulation of various types of particles [20,21,22,23,24]. Opposite to the attraction of magnetic particles towards a magnetic source where the magnetic field is the highest, diamagnetic (also called non-magnetic in many places) particles are repelled from the magnet if they are suspended in a magnetic fluid [25]. This article aimed to provide a comprehensive review of the reported studies, particularly recent advances, in diverse manipulations of diamagnetic particles in the flow of magnetic fluids. Before doing that, we first present a brief overview of the status for both magnetic particles and magnetic fluids. 

### 1.1. Magnetic Particles

Magnetic micro/nanoparticles have found wide applications in biomedicine, such as magnetic resonance imaging (MRI) contrast enhancement, hyperthermia, drug delivery, and magnetic separation [26,27,28]. The last two applications are both associated with the positive magnetophoretic motion of magnetic particles in response to magnetic field gradients. Specifically, a drug attached to a biocompatible magnetic carrier can be guided through the circulatory system by external high-gradient magnetic fields and delivered to a specific target site within the body [29]. Magnetic separation makes use of the magnetic field-induced deflection and/or retention of magnetic particles to separate them from non-magnetic particles [30,31]. This method has been demonstrated to sort target cells out of a heterogeneous mixture through either the labeling of functionalized magnetic beads [32,33,34] or the intrinsic paramagnetic feature [35]. Two examples of such separations are magnetic-activated cell sorting (MACS) [36] and magnetic field-flow fractionation (MFFF) [37]. In addition, magnetic particles have been used as solid supports for bioassays such as immunoassays and DNA/RNA hybridization, because their plugs, featuring high-surface-to-volume ratios, can be easily formed and controlled by a magnetic field [38]. The synthesis, manipulation, transport, and detection of magnetic particles have been and are still the foci of current research activities on magnetics in microfluidics. This area has been reviewed in several recent articles including those from Pamme [14], Liu et al. [39], Gijs et al. [40], Tekin and Gijs [41], and Rikken et al. [42].

### 1.2. Magnetic Fluids

There are two types of magnetic fluids that are commercially available and commonly used in microfluidic applications. The first type is paramagnetic fluids which includes the aqueous solutions of manganese(II) chloride (MnCl_2_) and gadolinium(III) chloride (GdCl_3_), among others. They are transparent and, thus, allow straightforward visualization of particles without the need of fluorescent labeling. However, paramagnetic solutions often have a weak magnetic susceptibility [43]. Therefore, the salt concentration needs to be high in order to induce sufficient magnetophoretic particle motion which has been found to cause issues in biocompatibility [44]. Alternatively, strong magnet(s) (e.g., superconducting magnets [45]) may be used to provide large magnetic fields (and magnetic field gradients as well). Another approach is to bring magnets close to or even in contact with the particle suspension [46] such that strong magnetic field gradients can be created in the solution. In addition, on-chip magnetic (e.g., nickel or cobalt) microstructures may be used along with typical neodymium–iron–Boron (NdFeB) permanent magnets to locally boost the magnetic field gradients [47]. However, both of these designs greatly increase the difficulty for on-chip particle manipulation if a pair of magnets, no matter repulsive or attractive, has to be employed. This issue may be resolved through the use of mechanical setups, which will be revisited later in appropriate sections (see Section 4.1 and Section 5.1). Readers interested in magnetophoresis of micro/nanoparticles in paramagnetic solutions may refer to the review articles from Pamme [9] and Watarai’s group [20,21].

The other type of magnetic fluid is the so-called ferrofluid, which is a suspension of ferro- or ferrimagnetic single domain nanoparticles (made of magnetite, Fe_3_O_4_, and usually of 10 nm in diameter) in pure water or organic oil with coated surfactants to prevent agglomerations [48]. Since the invention in early 1960s, ferrofluids have found many industrial applications, such as liquid seals in hard disks, coolants in loud speakers, and friction reducers in automotive [49]. Their first use in microfluidics was probably the magnetic micropump reported in a study by Hatch et al. in 2001 [50]. Ferrofluids usually have an initial magnetic susceptibility that is several orders of magnitude larger than that of paramagnetic solutions. Therefore, regular permanent magnets and even small-current electric magnets normally suffice to control the motion of diamagnetic microparticles in ferrofluid flows. However, ferrofluids suffer from several issues like the accumulation of iron oxide nanoparticles in microchannels where the magnetic field is higher, and the non-transparency to optical imaging of particles and cells, etc. Moreover, the biocompatibility of ferrofluids is still a big concern, which is discussed later in this paper (see Section 7.1). Current research activities on continuous-flow particle manipulation in ferrofluids are focused upon two primary directions depending on if the external magnetic field is uniform or not. The first direction uses a uniform magnetic field (e.g., via a permanent magnet that is much larger in size than the microfluidic chip) to magnetize surface-patterned micromagnets or suspended magnetic particles. Strong magnetic field gradients can thus be generated around the magnetic microstructures or particles. This method has been demonstrated by Yellen’s group to both pattern/concentrate submicron (and even nanometer) diamagnetic particles [51,52,53,54,55] and as well assemble magnetic/diamagnetic microparticles [56,57] in stationary ferrofluids. The second direction, which is to be reviewed in this article, is to utilize the inherently non-uniform magnetic field around a (permanent or electric) magnet to exert a magnetic force (and a magnetic torque as well) on diamagnetic particles in the flow of ferrofluids for continuous manipulations. Readers may also refer to a few other recent review articles on this topic such as Hejazian et al. [14], Yang et al. [22], Zhao et al. [23], and Gao et al. [24].

The rest of this article is organized as follows. Section 2 presents the theoretical background of the magnetic fluid-induced magnetic force and torque on diamagnetic particles. Section 3 reviews the studies on diamagnetic particle deflection in the flow of magnetic fluids under either a non-uniform or a uniform magnetic field. Section 4, Section 5, Section 6 and Section 7 reviews, in order, the studies that demonstrate the use of diamagnetic particle deflection for the continuous-flow focusing (Section 4), enrichment (Section 5), separation (Section 6), and medium exchange (Section 7) of diamagnetic particles, respectively. Section 8 concludes this article with the author’s personal perspectives of some future research directions in the field.

## 2. Theoretical Background 

### 2.1. Magnetic Force and Translation

In a non-uniform magnetic field, a particle (either magnetic or diamagnetic) experiences a magnetic force, $F_{m}$, as long as its magnetic polarizability is different from that of the suspending fluid. For a spherical particle, $F_{m}$ is given:

(1) $$F_{m}=\mu_{0}V_{p}\left[\left(M_{p}-M_{f}\right)\cdot \nabla \right]H$$

where $V_{p}$ is the particle volume, $\mu_{0}$ is the permeability of free space, $M_{p}$ is the particle magnetization, $M_{f}$ is the fluid magnetization, and H is the magnetic field at the particle center [58,59]. Analogous to the dielectrophoretic force induced by a non-uniform electric field [25,60], $F_{m}$ may direct a particle either towards (when $M_{p}-M_{f}>0$) or away from (when $M_{p}-M_{f}<0$) the magnetic source (where the magnetic field strength attains the maximum with ∇H>0) if the particle is more or less magnetically polarizable than the fluid. Note that Equation (1) is only valid when the variation of the magnetic field is negligible over the particle volume, which is satisfied in typical microfluidic applications with permanent magnets [58,59]. 

A paramagnetic solution is frequently treated as a linear magnetic fluid with $M_{f}=\chi_{f}H$, where $\chi_{f}$ (>0) is the solution’s magnetic susceptibility and is a positive function of its ionic concentration. Then, as a diamagnetic particle is also linearly polarizable (i.e., $M_{p}=\chi_{p}H$ with $\chi_{p}$ (<0)), being the particle’s magnetic susceptibility, $F_{m}$ in a paramagnetic solution can be approximately estimated from:

(2) $$F_{m\_PM}=\mu_{0}V_{p}\left(\chi_{p}-\chi_{f}\right)\left(H\cdot \nabla \right)H$$

If, however, the magnetic source is strong or the particle size is big such that the magnetic field variation over the particle volume becomes finite, a revised formula which considers the influence of particle magnetization may be used to replace Equation (2):

(3) $$F_{m\_PM}=3\mu_{0}\mu_{f}V_{p}\frac{\mu_{p}\;-\;\mu_{f}}{\mu_{p}\;+\;2\mu_{f}}\left(H\cdot \nabla \right)H=\mu_{0}\left(1+\chi_{f}\right)V_{p}\frac{3\left(\chi_{p}\;-\;\chi_{f}\right)}{3\;+\;\chi_{p}\;+\;2\chi_{f}}\left(H\cdot \nabla \right)H$$

where $\mu_{f}=1+\chi_{f}$ and $\mu_{p}=1+\chi_{p}$ are the permeability of the fluid and particle, respectively, and the fraction after the first equal sign (i.e., $\left(\mu_{p}-\mu_{f}\right)/\left(\mu_{p}+2\mu_{f}\right)$) resembles the Clausius–Mossotti factor in dielectrophoresis [61]. Apparently, Equation (3) reduces to Equation (2) if $\chi_{p}$ and $\chi_{f}$ are both much smaller than 1 (hence, $1+\chi_{f}\approx 1$ and $3+\chi_{p}+2\chi_{f}\approx 3$), which is reasonable for diamagnetic particles suspended in paramagnetic solutions [43]. 

In contrast, a ferrofluid behaves like a non-linear magnetic fluid unless the applied magnetic field is weak [48]. Moreover, its magnetization is usually orders of magnitude higher than that of a diamagnetic particle. Therefore, the magnetic force on a diamagnetic particle suspended in a ferrofluid is given by:

(4) $$F_{m\_FF}=-\mu_{0}V_{p}\left(M_{f}\cdot \nabla \right)H$$

(5) $$M_{f}=c_{f}M_{np}L\left(\beta \right)=c_{f}M_{np}\left(\cot h\left(\beta \right)-\frac{1}{\beta }\right)$$

(6) $$\beta =\frac{\mu_{0}V_{np}M_{np}H}{k_{B}T}$$

In the above, $c_{f}$ is the ferrofluid concentration or more accurately the volume fraction of the constitutive magnetic nanoparticles), $M_{np}$ is the saturation moment of these nanoparticles with $M_{np}$ being its magnitude, L(β) is the Langevin equation [48], β characterizes the ratio of magnetic energy (i.e., $\mu_{0}V_{np}M_{np}H$) to thermal energy (i.e., $k_{B}T$), $V_{np}$ is the volume of magnetic nanoparticles, H is the magnetic field strength, $k_{B}$ is the Boltzmann constant, and T is the fluid temperature. Recognizing that $M_{f}$ is collinear with the local magnetic field, $F_{m\_FF}$ in Equation (4) can be rewritten as:

(7) $$F_{m\_FF}=-\frac{\mu_{0}V_{p}c_{f}M_{np}L\left(\beta \right)}{H}\left(H\cdot \nabla \right)H$$

which is a strong function of particle size, ferrofluid concentration, and magnetic field (gradient).

In principle, the non-uniform magnetic field in Equation (1) (and as well its derivatives in either a paramagnetic solution or a ferrofluid) should be solved from the magnetic flux conservation equation,

(8) $$\nabla \cdot \left[\mu_{0}\left(M+H\right)\right]=0$$

where the material magnetization, M, is equal to $M_{f}$ within the magnetic fluid while becoming zero in both the microchannel substrate and the free space surrounding the magnet. In most magnetofluidic applications [10,17,22], we may safely neglect the magnetic fluid’s disturbance to the magnetic field (where Equation (8) is reduced to ∇·H=0) unless the fluid is highly concentrated. As such, we may use Furlani’s analytical formulae to obtain the magnetic field components in three dimensions [62]. However, if significant variations of magnetic fluid concentration occur due to, for example, the redistribution of magnetic nanoparticles in a ferrofluid/water co-flow through a microchannel adjacent to a magnetic source [63,64,65,66,67], Equation (8) will have to be used to determine the magnetic field in the magnetic fluid.

The magnetic force on a neutrally buoyant particle is balanced by the viscous drag of the suspending fluid. The resulting magnetophoresis causes a particle translation relative to the fluid (either at rest or in motion). This motion is called negative magnetophoresis for diamagnetic particles in a magnetic fluid (i.e., directed away from the magnetic source) and positive magnetophoresis for magnetic particles in a diamagnetic or less magnetic fluid (i.e., directed towards the magnetic source) [68]. It is usually much weaker than the primary flow speed in typical magnetofluidic applications. Hence, the velocity of particle magnetophoresis, $U_{m}$, with respect to the fluid flow can be obtained by balancing the magnetic force, $F_{m}$, in Equation (1) with Stokes’ drag via Newton’s 2nd law:

(9) $$m_{p}\frac{dU_{m}}{dt}=F_{m}-3\pi \eta aKU_{m}$$

where $m_{p}$ is the particle mass, t is the time coordinate, η is the fluid viscosity, a is the particle diameter, and K is the correction factor for Stokes’ drag because of the boundary effects [69]. Assuming the particle is initially at rest, one can easily solve Equation (9) for the magnetophoretic particle velocity:

(10) $$U_{m}=\frac{F_{m}}{3\pi \eta aK}\left(1-e^{-\frac{3\pi \eta aK}{m_{p}}t}\right)$$

Note that the exponential term in Equation (10) describes the acceleration phase of the particle, which has a characteristic time of $\tau_{p}=m_{p}/3\pi \eta aK=\rho_{p}a^{2}/18\eta K$. For a particle with a diameter of a=10 µm and a density of $\rho_{p}=1000$ kg/m^3^, $\tau_{p}$ is on the order of 10 µs and much smaller than the time scale of observation in typical microfluidic experiments. Therefore, particle magnetophoresis can be safely assumed to undertake the following terminal velocity at any time instant,

(11) $$U_{m}=\frac{F_{m}}{3\pi \eta aK}=\frac{\mu_{0}a^{2}\left[\left(M_{p}-M_{f}\right)\cdot \nabla \right]H}{18\eta K}$$

For particles with dissimilar (either larger or smaller) densities to the suspending magnetic fluid, the magnetic force may also be balanced by the buoyant force on a particle, which, for a diamagnetic particle in a paramagnetic solution in the absence of flow, is expressed as:

(12) $$\left(\rho_{p}-\rho_{f}\right)g=\mu_{0}\left(\chi_{p}-\chi_{f}\right)\left(H\cdot \nabla \right)H$$

where $\rho_{f}$ is the fluid density, and g is the gravitational acceleration. Thus, the particle density may be experimentally determined using a custom-designed magnetic field. This so-called magnetic levitation technique [43] has been demonstrated to measure the density of diamagnetic particles and cells as well as separating them by density in a stationary paramagnetic solution. We will not review here the fundamentals and applications of this flow-free particle manipulation technique. Readers interested in this topic may refer to the recent review articles from Turker and Arslan-Yildiz [70], Gao et al. [24], and Ge et al. [71] for more details. 

Because of the three-dimensional (3D) feature of magnetic field in the spatial coordinates, particle magnetophoresis, $U_{m}$, usually has both a streamwise and a cross-stream component in a confined fluid flow. The cross-stream magnetophoretic motion directs a particle towards the channel wall(s) that is either nearer to (positive magnetophoresis) or farther from (negative magnetophoresis) the magnet, which competes with the fluid flow leading to a transverse deflection of the particle (Section 3). Such a phenomenon has been be utilized to focus (Section 4), separate (Section 6), and wash (Section 7) diamagnetic particles and cells in magnetic fluids for various microfluidic applications. The streamwise magnetophoretic motion may be either along with or against the fluid flow, the latter of which has been utilized to overcome the advection of fluid flow for a continuous trapping and enrichment of particles (Section 5).

### 2.2. Magnetic Torque and Rotation

A particle also experiences a magnetic torque, $T_{m}$, in a magnetic field if it has a different magnetic polarizability from the suspending fluid:

(13) $$T_{m}=\mu_{0}\int \left[\left(M_{p}-M_{f}\right)\times H\right]dV$$

Dissimilar to the magnetic force, $T_{m}$ exists even in a uniform magnetic field, $H_{0}$, which, if the particle and fluid are both homogeneous, isotropic, and linearly magnetizable, is given by:

(14) $$T_{m}=\mu_{0}V_{p}\left(\chi_{p}-\chi_{f}\right)H^{-}\times H$$

In this equation, $H^{-}$ is the uniform magnetic field inside the particle and is a function of the particle aspect ratio as well as the fluid and particle’s magnetic properties [72,73]. Zhou et al. [74] obtained an expression for the magnetic torque in the x−y plane (i.e., about the z axis), $T_{mz}$, on an ellipsoidal particle:

(15) $$T_{mz}=-\frac{1}{2}\mu_{0}V_{p}{\left(\chi_{p}-\chi_{f}\right)}^{2}H_{0}^{2}\frac{\left(D_{yy}-D_{xx}\right)\sin2\left(\phi -\alpha \right)}{\left[1\;+\;\chi_{f}\;+\;\left(\chi_{p}-\chi_{f}\right)D_{xx}\right]\left[1+\chi_{f}+\left(\chi_{p}-\chi_{f}\right)D_{yy}\right]}$$

where $D_{xx}$ and $D_{yy}$ are the diagonal components of the demagnetizing factor, ϕ is the particle orientation with respect to the y-axis, and α is the magnetic field direction relative to the y-axis. For a prolate ellipsoid, $D_{xx}=1-A$ and $D_{yy}=A/2$, where $A=r_{p}^{2}/\left(r_{p}^{2}-1\right)-r_{p}{\cos h}^{-1}r_{p}/{\left(r_{p}^{2}-1\right)}^{3/2}$ denotes the elliptical integral with $r_{p}$ being the particle aspect ratio [75].

Therefore, a spherical particle, for which $r_{p}=1$ and, hence, A=2/3, does not experience a magnetic torque in a uniform magnetic field because of $D_{xx}=D_{yy}=1/3$. The magnetic torque has the same sign for both a diamagnetic particle in a magnetic fluid and a paramagnetic particle in a diamagnetic fluid because of its dependence on ${\left(\chi_{p}-\chi_{f}\right)}^{2}$. It causes a rotation of the non-spherical particle, which, in the absence of the particle inertial and wall effects, has an angular velocity, $\omega_{m}$ [76],

(16) $$\omega_{m}=-\frac{\mu_{0}H_{0}^{2}}{\eta }{\left(\chi_{p}-\chi_{f}\right)}^{2}\sin2\left(\phi -\alpha \right)\lambda \left(r_{p},\chi_{p},\chi_{f}\right)$$

where $\lambda \left(r_{p},\chi_{p},\chi_{f}\right)$ is a function of the particle and fluid properties. This magnetic rotation is additive to the traditional hydrodynamic rotation, the result of which leads to a shape-dependent lateral migration of non-spherical particles in a confined fluid flow [74]. It is important to note that the above-presented formulae for the magnetic force and torque as well as the resulting magnetic translation and rotation are all valid for single particles only. The magnetic (as well as hydrodynamic) interactions among particles will have to be considered if the particle concentration is not sufficiently low. 

## 3. Particle Deflection

The extent of particle deflection in a magnetic fluid flow scales with the ratio of the particle velocities perpendicular (e.g., in the y− or z-direction) and parallel to the fluid velocity, $U_{f}$, in the x-direction:

(17) $$deflection_{i}\propto \frac{U_{m,i}}{U_{m,x+U_{f}}}$$

where $U_{m,i}$ (i=x, y or z) is the velocity component of particle magnetophoresis in each of the three directions. Referring to Equation (11), we see that the magnetic deflection of a spherical particle is a strong function of the particle and magnetic fluid properties as well as the magnetic field (including both the strength and the gradient). The former dependence enables the continuous-flow magnetic separation of particles by size or magnetic susceptibility without any labeling, which is reviewed later in Section 6. We present below the published papers on diamagnetic particle deflection in the flow of magnetic fluids under an externally imposed non-uniform (Section 3.1) or uniform (Section 3.2) magnetic field. 

### 3.1. Non-Uniform Magnetic Field

**Figure 1:**
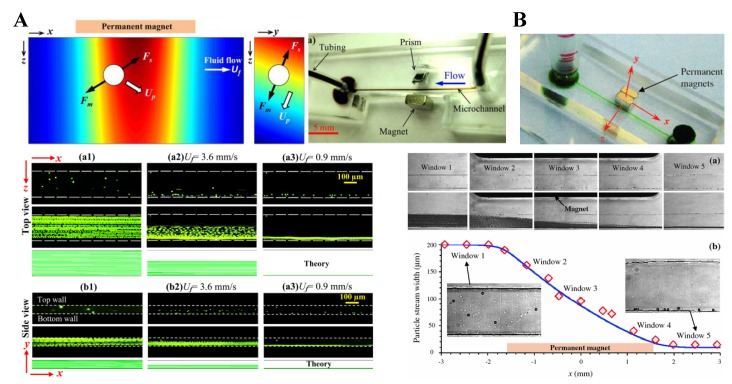
Diamagnetic particle deflection in the flow of magnetic fluids under a non-uniform magnetic field. (**A**) Force analyses on a diamagnetic particle in the flow of magnetic fluids in the horizontal (left) and vertical (top left) planes of a microchannel, where the background colors show the magnetic field contour; Experimental and theoretical demonstrations (bottom) of the deflection of 5 µm diamagnetic beads in 0.5× EMG 408 ferrofluid through a straight rectangular microchannel with a nearby permanent magnet (top right). Adapted with permission from Liang et al. [79], © 2011 American Institute of Physics. (**B**) Experimental and theoretical studies (bottom) of the lengthwise development for the deflection of 10 µm diamagnetic beads in 1M MnCl_2_ solution with two stacked permanent magnets (top). Adapted with permission from Zhu et al. [80], © 2012 Springer.

Tarn et al. [45] described a microfluidic system for continuous-flow particle separation via the diamagnetic repulsion from a high magnetic field. This system is in principle analogous to that of on-chip free flow magnetophoresis, where magnetic particles can be separated from each other and diamagnetic particles while they are attracted towards the high magnetic field [77,78]. The authors studied the deflection behaviors of 5 µm and 10 µm diamagnetic PS beads in 6% and 10% aqueous solutions of paramagnetic MnCl_2_ in the non-uniform magnetic field of a superconducting magnet. Under a constant flow rate of 400 µL/h, they observed a greater deflection for larger beads in a higher concentration of MnCl_2_, which is consistent with the prediction of Equation (11). Such a diamagnetic particle deflection takes place in both directions of the channel’s cross-sectional plane because of the intrinsic magnetic field gradients created by a permanent magnet (Figure 1A, top left) [79]. The result is a tight particle stream flowing near the bottom outer corner of the microchannel that is the farthest to the magnet. Liang et al. [79] demonstrated this deflection phenomenon in EMG 408 ferrofluids (Ferrotec) through a real-time imaging of both the horizontal and vertical plane of the microchannel (Figure 1A, top right). They also developed a 3D analytical model to simulate the diamagnetic particle deflection behaviors in ferrofluid flows. Their model predicts with a good agreement the effects of multiple parameters (Figure 1A, bottom). In another study, Zhu et al. [80] studied the deflection of diamagnetic PS beads in MnCl_2_ solutions through a straight rectangular microchannel. They developed an easy approach to embedding permanent magnets into poly(dimethylsiloxane) (PDMS)-based microfluidic chips with a 260 µm magnet-channel distance (Figure 1B, top). Hence, micro beads of blood cell sizes (i.e., 5–15 µm in diameter) can be effectively manipulated in typical paramagnetic solutions without the use of a superconducting magnet (Figure 1B, bottom). 

**Figure 2:**
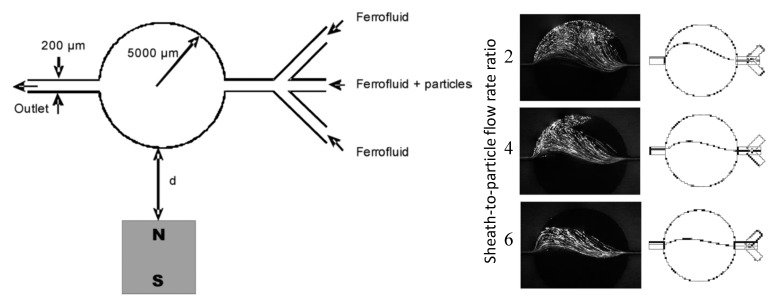
Deflection of pre-focused diamagnetic particles in magnetic fluids under a non-uniform magnetic field: Schematic of the microchannel/magnet setup (left); Comparison of the experimentally and numerically obtained deflections of diamagnetic beads in a diluted EMG 707 ferrofluid (Ferrotec) inside the circular chamber at varying flow rate ratios (right). Adapted with permission from Hejazian and Nguyen [83], © 2015 The Royal Society of Chemistry.

There are a couple of other reports on the deflection of pre-focused diamagnetic particles in the flow of magnetic fluids. Zhu et al. [81] presented a two-dimensional (2D) analytical model and as well the experimental verification for the transport of PS beads in ferrofluid flow through a straight rectangular channel section with a nearby permanent magnet. The beads were pre-focused to the mid-plane of the channel by two equal flows of the suspending ferrofluid, such that they were deflected across the ferrofluid stream from the same starting position. This arrangement significantly reduces the computational time in predicting the particle trajectories. They experimentally studied the effects of both bead size and flow rate on the trajectories of beads, which confirmed the validity of their analytical model. Later, Cheng et al. [82] extended the analytical model from 2D to 3D. In another study, Hejazian and Nguyen [83] studied the magnetic deflection of pre-focused diamagnetic beads in a similar setup to Zhu et al. [81], except that the beads and ferrofluids were introduced into a circular chamber (Figure 2, left). They observed various working regimes with the change of the sheath flow rate (the particle flow rate was fixed), bead size, and magnet-chamber distance. These observed phenomena were not fully captured by their numerical simulation of bead trajectories, especially at the high sheath-to-particle flow rate ratio (Figure 2, right). The authors attributed the deviations to the magnetically induced secondary flow in the ferrofluid, which grows stronger for a smaller sheath flow rate and under a greater magnetic field. 

### 3.2. Uniform Magnetic Field

**Figure 3:**
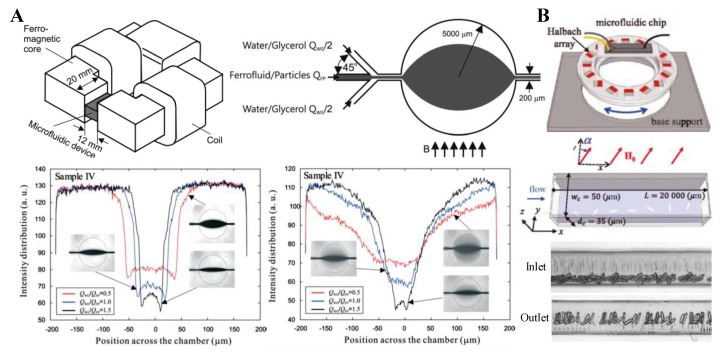
Diamagnetic particle deflection in the flow of magnetic fluids under a uniform magnetic field. (**A**) Experimental setup for the study of migration of diamagnetic beads in a water-sandwiched ferrofluid core flow through a circular chamber in the uniform magnetic field of an electromagnet (top); bead migration-induced ferrofluid spreading (by images and intensity profiles) across the circular chamber when the magnetic field is off and on (bottom). Adapted with permission from Zhu et al. [84], © 2014 The Royal Society of Chemistry. (**B**) Schematic illustration of the microfluidic chip (top) and straight microchannel (middle) in the uniform magnetic field of a Halbach array; superimposed images illustrating the magnetic torque-induced lateral migration of pre-focused ellipsoidal diamagnetic beads in a ferrofluid with a uniform magnetic field perpendicular to the flow (bottom). Adapted with permission from Zhou et al. [74], © 2017 American Physical Society.

Equation (11) indicates that the magnetophoretic motion of spherical particles ceases if the externally imposed magnetic field becomes uniform. There are, however, two exceptions to this circumstance. One is for particles in a flow that has multiple fluids with non-matching magnetic properties, where magnetic field gradients appear at the fluid interface because of the non-uniform fluid magnetization (see Equation (8)). Zhu et al. [84] verified this analysis through an experimental study of the migration of diamagnetic beads in a ferrofluid core flow that is sandwiched between two diamagnetic water/glycerol streams in the uniform magnetic field of an electromagnet (Figure 3A, top). They observed a strongly enhanced spreading of the ferrofluid across the circular chamber because of the negative magnetophoretic motion of the suspended 1 µm PS beads towards the diamagnetic sheath fluids (Figure 3A, bottom). The other exception happens if the diamagnetic particle is not spherical, where a magnetic torque (see Equation (13)) is induced that can alter the rotation dynamics of the particle and results in a shape-dependent lateral migration in a confined flow even under a uniform magnetic field. Zhou et al. [74] experimentally investigated such a lateral migration for ellipsoidal diamagnetic and paramagnetic beads in the flow of a ferrofluid and a diamagnetic water/glycerol solution, respectively, under the uniform magnetic field of a custom-made Halbach array (Figure 3B, top). By varying the direction of the magnetic field with respect to that of the flow (i.e., α in Equation (13) and Figure 3B, middle), the authors demonstrated a diverse control of the symmetric and asymmetric rotations of non-spherical beads. As a result, the beads can migrate towards either the center (Figure 3B, bottom) or the walls of the microchannel. 

### 3.3. Summary

Table 1 summarizes the above-reviewed experimental studies on the deflection of diamagnetic particles in the flow of magnetic fluids. To facilitate reading, we have included in the table the parameters for particle size, magnetic fluid concentration, and particle flow rate (excluding the sheath flow rate if any) as well as the magnet setup (note these parameters will also be included in Tables 2–5 in the following sections). 

## 4. Particle Focusing

Particle focusing refers to the alignment of particles into either a 2D plane (and, hence, a one-dimensional (1D) focusing) or a 1D line (and, hence, a 2D focusing) at the center(plane) of a microchannel. This operation is a crucial step prior to the detection and analysis of single particles for various industrial and biomedical applications [85,86]. It has been realized in microfluidic devices by the use of a variety of force fields, which may be externally applied such as the electric, acoustic, and optical forces, etc. or internally induced, such as the inertial and elastic forces, etc. The most powerful particle focusing method is still the use of two or more sheath flows, which remains effective regardless of the particle size. Readers are suggested to refer to a few recent review articles on this topic such as Xuan et al. [87], Martel and Toner [88], and Zhang et al. [89]. Below we first review the works on diamagnetic particle focusing in the flow of magnetic fluids using a pair of magnets (Section 4.1). We then review those using only a single magnet, where a sheath flow may be used to assist the particle focusing (Section 4.2) or is not needed (Section 4.3).

### 4.1. Repulsing Magnet Pair

**Figure 4:**
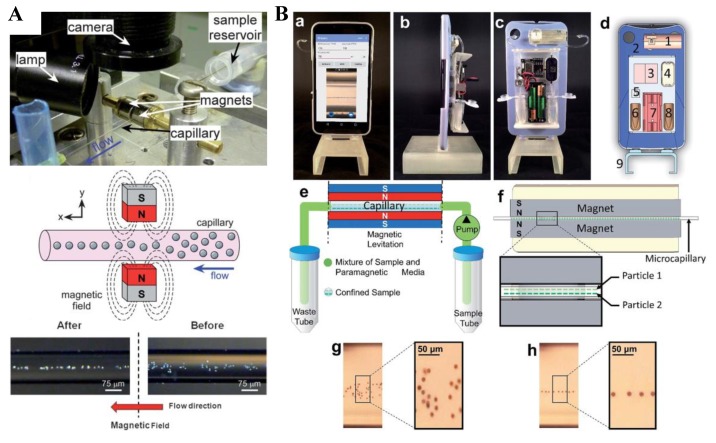
Diamagnetic particle focusing in the flow of paramagnetic solutions under the magnetic field of a pair of repulsing magnets. (**A**) Experimental setup (top) of a capillary positioned between a pair of mechanically fixed permanent magnets (middle) and images for the focusing of 10 µm PS beads into the capillary center at an average flow velocity of 670 µm/s (bottom). Adapted with permission from Rodriguez-Villarreal et al. [44], © 2011 The Royal Society of Chemistry. (**B**) Smart-phone attachable, flow-assisted diamagnetic particle focusing device (top) with a glass square capillary positioned between a mechanically fixed magnet pair (middle); distribution of 10 µm PS beads flowing through the capillary at different flow rates (bottom). Adapted with permission from Amin et al. [91], © 2016 The Royal Society of Chemistry.

Pamme’s group [90] were among the first to utilize the repulsion force from a high magnetic field to focus diamagnetic particles in a continuous flow of paramagnetic solutions. Rodriguez-Villarreal et al. [44] suspended diamagnetic beads in a MnCl_2_ solution or cells in a GdCl_3_ solution and pumped the suspension through a cylindrical capillary between a pair of mechanically fixed permanent magnets with the like poles facing each other (i.e., a repulsing magnet pair) (Figure 4A, top and middle). The beads or cells were repelled by each magnet and aligned into the central axis of the capillary for a 2D focusing (Figure 4A, bottom). This diamagnetic particle focusing was greatly enhanced with increased medium susceptibility or particle size and reduced flow rate. Based on a similar principle, Amin et al. [91] developed a smart-phone attachable continuous flow diamagnetic particle focusing device (Figure 4B, top). Diamagnetic PS beads of 10 µm in diameter were suspended in a paramagnetic 50 mM Gadavist solution, and pumped through a glass square capillary located between two permanent magnets in the horizontal slots (Figure 4B, middle). At an appropriate flow rate, all beads were confined at a similar vertical equilibrium height because of the balance of the gravity with the diamagnetic repulsion force (Figure 4B, bottom). Moreover, a magnifying lens was placed between the smart-phone camera and capillary to capture particle images, and a custom-developed Android application was used for both imaging control and communications.

**Figure 5:**
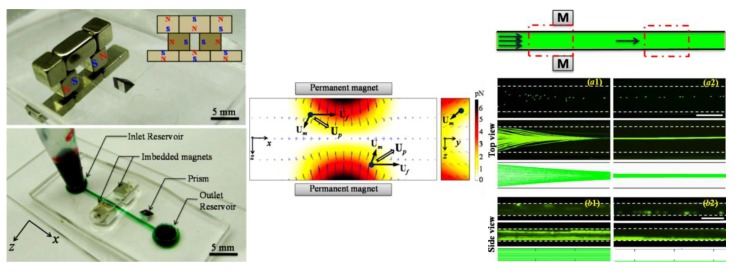
Diamagnetic particle focusing in ferrofluid flows under the magnetic field of a pair of repulsing magnets: Picture of the placed magnets/prism (upper left) for fabricating the microfluidic device (lower left); Velocity analysis of diamagnetic particles in the flow of ferrofluid in the horizontal and vertical planes of the microchannel (middle); Experimental and theoretical images of the 2D focusing (i.e., both horizontal and vertical) of 5 μm diamagnetic beads before and after the magnet pair (right). Adapted with permission from Zeng et al. [93], © 2012 IOP Publishing Ltd.

There are a couple of other studies on the focusing of diamagnetic particles in the flow of ferrofluids under the magnetic field of a repulsing magnet pair. Zhu et al. [92] incorporated permanent magnets into the microfluidic device during the PDMS curing step. They were able to embed repulsing magnets into the opposite sides of a straight rectangular microchannel with a small gap, thereby creating large magnetic field gradients across the channel. The authors studied the magnetic focusing of 4.8 µm, 5.8 µm, and 7.3 µm PS beads in EMG 408 ferrofluid at various flow rates in the horizontal plane of the microchannel. They also extended the 2D analytical model developed in their earlier work [81] to simulate the 3D bead trajectories during the focusing process. In a later study, Zeng et al. [93] revised the approach to embedding repulsing permanent magnets into the PDMS layer symmetrically about a straight rectangular microchannel (Figure 5, upper left). The edge-to-edge distance between the magnets was determined solely by the size of the magnets themselves. The device that the authors developed (Figure 5, lower left) was capable of focusing 5 μm diamagnetic beads and yeast cells into tight streams flowing near the centerline of the bottom channel wall in a diluted EMG 408 ferrofluid (Figure 5, right) because of the large magnetic field gradients in both the horizontal and vertical planes of the microchannel (Figure 5, middle). The authors also developed a 3D analytical model to predict the observed particle and cell focusing behaviors at various conditions (Figure 5, right). 

### 4.2. Single Magnet with a Sheath Flow

**Figure 6:**
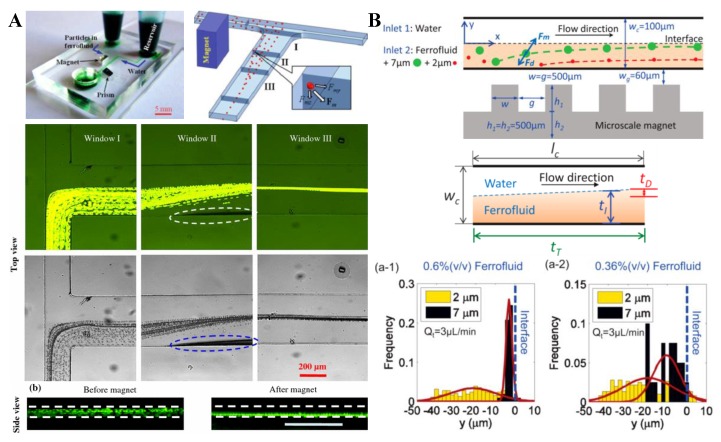
Sheath flow-assisted diamagnetic particle focusing in ferrofluid flows under the magnetic field of a single permanent magnet. (**A**) Picture of the microfluidic device and schematic illustration of the diamagnetic particle focusing mechanism in a T-shaped microchannel (top); top-view (both in fluorescent and bright field) and side-view images of the horizontal and vertical focusing of 5 µm diamagnetic beads in 0.01× EMG 408 ferrofluid (bottom). Adapted with permission from Liang et al. [94], © 2012 Springer. (**B**) Enlarged view of the microchannel ferrofluid particle micro-magnet system (top) and illustration of the time scales for particle migration in a two-phase flow system (middle); plots for the Gaussian distribution of 2 µm and 7 µm diamagnetic beads at the channel outlet (bottom). Adapted with permission from Zhou and Wang [95], © 2016 American Institute of Physics.

Considering the difficulty of positioning closely a pair of repulsive magnets in the above-presented particle focusing techniques, Liang and Xuan [94] developed a new approach to focusing diamagnetic particles in ferrofluids with a single permanent magnet. In a co-flow of ferrofluid suspension and water through a T-shape microchannel, particles are deflected across the ferrofluid by negative magnetophoresis and confined by the diamagnetic water flow, achieving a 2D focusing near the centerline of the bottom channel wall (Figure 6A, top). Superior to the traditional two-magnet configuration [90,91,92,93], the single magnet can be placed close enough to the microchannel such that the opaque ferrofluid may be sufficiently diluted for a bright-field visualization of the suspended particles without labeling. The authors demonstrated the technique by studying the focusing of 5 µm and 10 µm PS beads in 0.01× EMG 408 ferrofluid (i.e., 100 times dilution) in both the horizontal (top view) and vertical (side view) planes of the microchannel (Figure 6A, bottom). A similar idea was later implemented by Zhou and Wang [95], who exploited the easily fabricated microscale magnet to generate even stronger magnetic field gradients and forces inside the microchannel (Figure 6B, top). They did an analysis of the time scales (Figure 6B, middle) and pointed out that the ferrofluid/water interface remains sharp and stable because of the high Peclet number associated with the flow. They used 2 µm and 7 µm PS beads in slightly diluted EMG 408 ferrofluids, where the larger beads were observed to achieve an apparently better focusing (Figure 6B, bottom). 

### 4.3. Single Magnet Only

**Figure 7:**
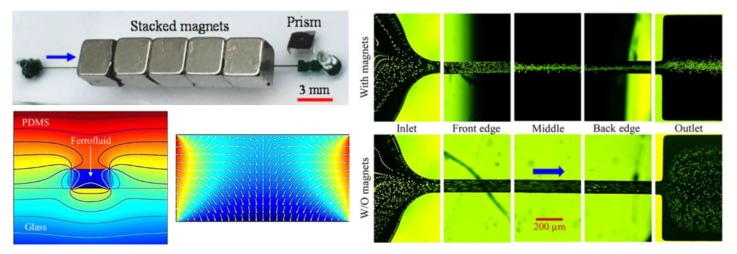
Sheath-free diamagnetic particle focusing in the flow of ferrofluids under the magnetic field of a single magnet: Picture of the microfluidic chip with 5 stacked permanent magnets right above (upper left); Simulated magnetic field contour and magnetic force in the cross-section the device (lower left); Experimental images of 10 μm diamagnetic beads in the 0.75 mL/h flow of 0.75× EMG 408 ferrofluid through a straight rectangular microchannel with and without the magnets (right). Adapted with permission from Chen et al. [97], © 2018 American Chemical Society.

There are also two studies that each reported a sheath-free diamagnetic particle focusing with a single permanent magnet. Yan et al. [96] proposed a new concept of magnetophoresis-assisted hydrophoretic focusing of diamagnetic particles using a single magnet on top of the microchip. In their design, diamagnetic particles suspended in a dilute ferrofluid were repelled to the lower level of the hydrophoretic channel by negative magnetophoretic force, and hence interact with the grooves of the microchannel more effectively to achieve an enhanced hydrophoretic ordering. The authors confirmed the advantages of magnetophoresis in such a hybrid particle focusing technique using a combined experimental and numerical study. They further investigated this magnetophoresis-assisted hydrophoretic focusing system using PS beads of various sizes in ferrofluids of varying concentrations in a range of flow rates. They achieved a high-throughput focusing at a flow rate as high as 3 mL/h. Chen et al. [97] developed a novel and simple method for 2D focusing of diamagnetic particles in a microchannel ferrofluid flow with a single set of overhead permanent magnets (Figure 7, upper left). Particles are focused by the diamagnetic repulsion force into a single stream near the bottom wall of a straight rectangular microchannel (Figure 7, right), where the magnetic field reaches the minimum because of the magnetization of the ferrofluid (Figure 7, lower left). Such a focusing can be readily switched on and off by replacing and removing the magnets. Moreover, the exiting position of the focused particles can be tuned by shifting the magnets relative to the microchannel. The authors also performed a systematic experimental study of various parametric effects on the focusing of PS beads in terms of a defined particle-focusing effectiveness.

### 4.4. Summary

Table 2 summarizes the above-reviewed studies on the continuous-flow focusing and alignment of diamagnetic particles using magnetic fluids.

**Table 2:** Summary of the published papers on diamagnetic particle focusing in magnetic fluid flows.

Sheath	Particles	Fluid	Magnet		Particle Flow Rate	References
			Configuration	Type		
-	10 µm PS	0.79 M MnCl_2_	repulsing pair	permanent	43 µL/h	[90]
-	10/20 µm PS HaCaT cells	0.79 M MnCl_2_/GdCl_3_	repulsing pair	permanent	30 µL/h	[44]
-	10 µm PS	50 mM Gadavist	repulsing pair	permanent	~500 µL/h	[91]
-	4.8/5.8/7.3 µm PS	1.2% ferrofluid	repulsing pair	permanent	60–480 µL/h	[92]
-	5 µm PS yeast cells	0.3% ferrofluid	repulsing pair	permanent	50 µL/h	[93]
water sheath	5/10 µm PS	0.012% ferrofluid	single	permanent	~50 µL/h	[94]
water sheath	2/7 µm PS	0.36/0.6% ferrofluid	single	permanent + micromagnet	90 µL/h	[95]
-	5–13 µm PS	0.024–0.12% ferrofluid	single	permanent	0.6–4.8 mL/h	[96]
-	5/10/20 µm PS	0.6–1.2% ferrofluid	stacked	permanent	0.1–2 mL/h	[97]

## 5. Particle Enrichment

Particle enrichment refers to the trapping and concentration of particles at designated spots, which is essentially a 3D focusing towards a point (in contrast to the above-noted 1D focusing towards a plane and 2D focusing towards a line; see Section 4). It is usually necessary for a reliable detection of low amount of particles in applications ranging from environment monitoring to food safety and water quality control etc. In microfluidic devices particle enrichment can take place in a contact or a contactless mode [98,99]. The contact method immobilizes particles of any kind onto a surface by mechanical blocking or only specific particle types by chemical adhesion [100], which is usually prone to irreversible particle attachment. The contactless method uses a force, which may be of any origin such as that from the application of an electric, acoustic, or optical field, to trap and concentrate particles in suspensions. It has the advantage of easy switching between the particle trapping and releasing modes. Readers are suggested to refer to a few review articles for recent progress in this direction [101,102,103]. We briefly present below the studies on diamagnetic particle enrichment in magnetic fluids under the magnetic field of either a pair of magnets (Section 5.1) or a single magnet (Section 5.2). 

### 5.1. Magnet Pair

**Figure 8:**
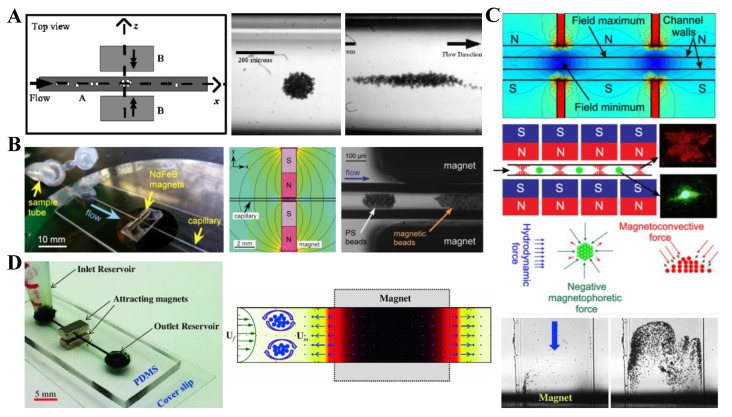
Diamagnetic particle trapping and enrichment in the flow of magnetic fluids under the magnetic field of a pair of permanent magnets. (**A**) Top-view schematic of the particle trapping region between two repulsing magnets (left); self-assembled sphere (middle) and ellipsoid (right) of 21 µm diameter PS beads in 0.005% ferrofluid using two opposing cylindrical and rectangular magnets, respectively. Adapted with permission from Feinstein and Prentiss [104], © 2006 American Institute of Physics. (**B**) Picture of the diamagnetic particle trapping setup (left) and simulated magnetic field of two attracting magnets (middle); simultaneous formation of a plug of 8 µm magnetic beads between the magnets and a plug of 10 µm diamagnetic beads before the magnet pair (right). Adapted with permission from Tarn et al. [105], © 2013 The Royal Society of Chemistry. (**C**) Simulated magnetic field illustrating the concept of magnetofluidic concentration of particles (top); schematic and demonstration of the size-selective traps for diamagnetic large green and small red beads (middle) with a schematic description of the dominant forces (bottom). Adapted with permission from Hejazian and Nguyen [106], © 2016 American Institute of Physics. (**D**) Picture of the microfluidic device sandwiched between two attracting magnets (left) and illustration of the diamagnetic particle trapping mechanism (middle); snapshot images showing the magnetic enrichment of 5 µm PS beads in 0.05× EMG 408 ferrofluid at 5 s and 5 min after the flow was switched on (right). Adapted with permission from Zeng et al. [107], © 2013 Springer.

Continuous-flow diamagnetic particle enrichment has thus far been demonstrated with both a repulsing and an attracting magnet pair. There is only one report on the former, where Feinstein and Prentiss [104] developed an inexpensive and simple system for 3D self-assembly of diamagnetic particles into millimeter-scale structures in the flow of ferrofluid through a glass square capillary. They used two opposing permanent magnets to create a magnetic field zero at the center of the capillary for particle trapping (Figure 8A, left). They demonstrated the system by trapping and assembling 5 µm, 10 µm, and 21 µm diameter PS beads into millimeter-sized structures in 0.005% v/v EMG 509 ferrofluid (Ferrotec), which may be of spherical (Figure 8A, middle) or ellipsoidal (Figure 8A, right) shape depending on if circular or rectangular magnets were used. Their method allows assembling and maintaining structures in suspension without invasive labeling or attachment to a permanent solid surface. Peyman et al. [90] first demonstrated the use of diamagnetic repulsion to trap particles in a paramagnetic solution through a capillary positioned between two attracting magnets (Figure 8B, left). In their setup, the magnetic field attains the maximum between the two opposing faces of the magnets (Figure 8B, middle), whereby magnetic particles are attracted because of positive magnetophoresis. Outside of the magnets region, the magnetic field drops off sharply along the capillary, which generates a repulsion force for diamagnetic particles. Tarn et al. [105] demonstrated the application of these distinctive magnetic forces for the simultaneous trapping of 8 µm magnetic and 10 µm diamagnetic beads in the flow of 0.79 M MnCl_2_ via a single pair of magnets (Figure 8B, right). 

Later, Wilbanks et al. [108] studied the influence of magnetic asymmetry on the pattern and flow rate of 5 µm diamagnetic particle concentration in 0.2× EMG 408 ferrofluid. They found that breaking the symmetry of the magnets with respect to a straight rectangular microchannel changes the particle trapping from inside two symmetric counter-rotating circulations to a single asymmetric circulation. Moreover, the magnet asymmetry increases the maximum flow rate for a stable particle trapping, which was reasonably predicted by a 3D theoretical model. Hejazian and Nguyen [106] used a similar setup with two arrays of attracting permanent magnets for a size-selective concentration of diamagnetic particles in diluted ferrofluids. The magnets create multiple capture zones with minimum magnetic field strength along the rectangular channel (Figure 8C, top). The complex interactions among the magnetophoretic, magneto-convective, and hydrodynamic forces (Figure 8C, bottom) yield the enrichment of 3.1 µm and 4.8 µm PS beads into the magnetic field maxima and minima, respectively. In another study, Zeng et al. [107] developed a simple magnetic technique to concentrate 5 µm PS beads and yeast cells in a dilute ferrofluid flow through a straight rectangular microchannel using negative magnetophoresis. The magnetic field gradient was created by two attracting permanent magnets that were placed on the top and bottom of the planar microchip and held in position by their natural attractive force (Figure 8D, left). The authors analyzed the magnetic field and, hence, the induced magnetophoretic motion of particles (Figure 8D, middle). They demonstrated the dynamic development of a particle trapping pattern with time (Figure 8D, right). 

### 5.2. Single Magnet

**Figure 9:**
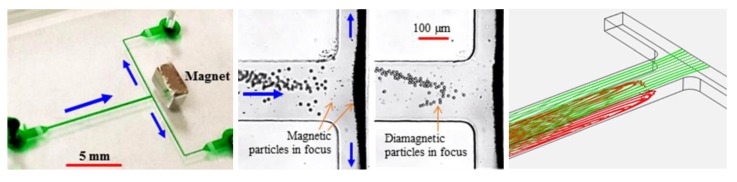
Diamagnetic particle enrichment in the flow of magnetic fluids through a T-shaped microchannel with a single permanent magnet: picture of the microfluidic chip (left), top-view snapshot images for the simultaneous trapping of 9.9 µm diamagnetic and 2.85 µm magnetic beads at the T-junction (middle), and isometric view of the numerically predicted 3D trajectories of diamagnetic (red) and magnetic (green) beads (right). Adapted with permission from Zhou et al. [109], © 2015 American Institute of Physics.

The above-presented methods for diamagnetic particle enrichment all require placing magnet pairs close to the particle suspension. The strong magnetic force, no matter repulsive (Figure 8A) or attractive (Figure 8C,D), between the magnets makes them difficult to align and place close enough to a microchannel. To resolve this issue, Zhou et al. [109] proposed the use a single permanent magnet for a simultaneous trapping of diamagnetic and magnetic beads in a diluted ferrofluid flow through a T-shaped microchannel (Figure 9, left). A permanent magnet was placed symmetrically at the top of the T-junction. Due to the induced negative and positive magnetophoresis, 9.9 µm diameter diamagnetic PS beads were trapped onto the bottom wall of the main-branch while 2.85 magnetic beads were concentrated into the top corner of the side-branch that was the nearest to the magnet (Figure 9, right). These selectively concentrated beads could be sequentially released from the T-junction by simply tuning the ferrofluid flow rate. The authors also developed a 3D numerical model, which predicts both the trajectories of diamagnetic/magnetic beads and the buildup of ferrofluid nanoparticles with a good agreement.

### 5.3. Summary

Table 3 summarizes the above-reviewed studies on the continuous-flow trapping and concentration of diamagnetic particles using magnetic fluids.

**Table 3:** Summary of the published papers on diamagnetic particle enrichment in magnetic fluid flows.

Particles	Fluid	Magnet		Particle Flow Rate	References
		Configuration	Type		
5/10/21 µm PS	0.005% ferrofluid	repulsing pair	permanent	0.24–1.2 mL/h	[104]
10 µm PS	0.79 M MnCl_2_	attracting pair	permanent	43 µL/h	[90]
10 µm PS 8 µm mag	0.79 M MnCl_2_	attracting pair	permanent	10 µL/h	[105]
5 µm PS	0.24% ferrofluid	attracting pair	permanent	100–200 µL/h	[108]
5 µm PS yeasts	0.06% ferrofluid	attracting pair	permanent	240 µL/h	[106]
3.1/4.8 µm PS	0.005–1% ferrofluid	attracting array	permanent	0.6–6 mL/h	[107]
9.9 µm PS 2.85 µm mag	0.06% ferrofluid	single	permanent	~50 µL/h	[109]

## 6. Particle Separation

Particle separation refers to the sorting of particles from a heterogeneous mixture based on either an extrinsic label (physical or chemical for specificity) or an intrinsic property (such as size, density, shape, etc.). This operation is important for a wide range of applications that require purified particles in industry, biology, and medicine, etc. [110,111,112,113]. It has been demonstrated in microfluidic devices using a variety of active (e.g., driven by an electric [114], acoustic [115], or optical force [116]) and passive (e.g., via the inertial and/or elastic focusing [117,118,119]) methods. Readers are suggested to refer to recent review articles on this subject (e.g., [120,121,122], to name just a few). We review below the published papers on continuous separation of diamagnetic particles in magnetic fluids, which are divided into groups based on how the particles are focused prior to the separation. We will first review the studies that use either one sheath flow (Section 6.1) or two sheath flows (Section 6.2) to pre-focus diamagnetic particles in the magnetic fluid. Following those are the studies not using any sheath flows, which are divided into the sub-groups of sheath free (Section 6.3) and hybrid (Section 6.4) for the purpose of highlighting the latter. 

### 6.1. One-Sheath-Flow Focusing

**Figure 10:**
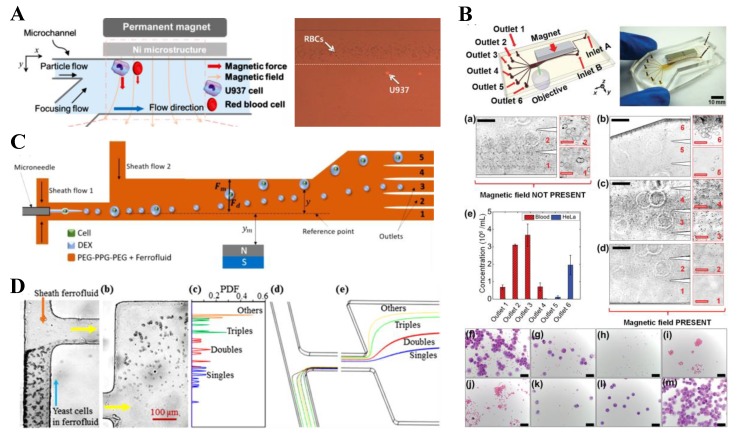
Diamagnetic particle separation in the flow of magnetic fluids with one-sheath-flow focusing. (**A**) Schematic of the separation mechanism (left) and the microscopic image of U937 cells separated from red blood cells (RBC) in 40 mM Gd−DTPA (right). Adapted with permission from Shen et al. [127], © 2012 American Chemical Society. (**B**) Schematic and picture of the microfluidic chip (top) and experimental images demonstrating the separation of HeLa and RBC cells (bottom). Adapted with permission from Zhao et al. [128], © 2015 Wiely-VCH. (**C**) Schematic illustration of diamagnetic separation of cell-containing droplets from empty droplets in the flow of ferrofluid with surfactants. Adapted with permission from Navi et al. [129], © 2018 The Royal Society of Chemistry. (**D**) Experimental (left) and numerical (right) demonstrations of ferrofluid-based magnetic fractionation of drug treated yeast cells in a T-shaped microchannel. Adapted with permission from Chen et al. [130], © 2017 American Institute of Physics.

Pamme’s group [90] first proposed the concept of free-flow diamagnetophoresis, which, similar to the traditional free-flow magnetophoresis [123,124], utilizes a perpendicular magnetic field to separate sheath-flow focused diamagnetic particles based on size or other intrinsic properties (e.g., shape) in the flow of paramagnetic solutions. Considering the relatively small magnetic susceptibility of paramagnetic solutions, Vojtisek et al. [125] proposed the incorporation of a microfluidic chip into a superconducting magnet for precise controls of objects in high magnetic fields. They used this system to demonstrate a label-free separation of a MnCl_2_-stream focused mixture of 5 µm and 10 µm polymeric beads in 0.48 M MnCl_2_ solution via diamagnetic repulsion. Kawano and Watarai [126] employed a similar idea to construct a 2D micro-flow magnetophoresis device in a superconducting magnet using triangular shaped pole pieces. They demonstrated a continuous-flow ternary separation of 1, 3 and 6 µm PS beads in 1 M MnCl_2_ solution. Another work that used a paramagnetic solution to generate the diamagnetic repulsion force for separation is from Shen et al. [127], who utilized a nickel microstructure (Figure 10A, left) to boost the magnetic field gradients in the fluid. The authors demonstrated an effective separation of both PS beads and biological cells (Figure 10A, right) based on the difference in size in 40 mM Gd-diethylenetriaminepentaacetic acid (DTPA) solution. 

There are multiple other studies that used ferrofluid as the suspending medium of particles for an enhanced diamagnetic repulsion force. Mao’s group made significant contributions in this direction. In their first paper of the series, Zhu et al. [131] demonstrated the binary separation of 1, 1.9 or 3.1 µm PS beads from 9.9 µm ones in the flow of EMG 408 ferrofluid through a straight rectangular microchannel with two-inlets and two outlets. They later used a similar device to separate by size *E. coli* cells/7.3 µm beads, yeast cells/1.0 beads, and yeast/*E. coli* cells. They found that the commercial water-based ferrofluid, EMG 408, was not detrimental to the viability of both cell types after a 2 h exposure [132]. Zhao et al. further revised the device design by increasing the number of outlets to six (Figure 10B, top). They also customized ferrofluids for label-free biocompatible separation of HeLa and red blood cells (Figure 10B, bottom) [128] as well as the high-throughput separation of cancer cells from white blood cells (WBCs) [133]. Another work worthy of notice is the continuous separation of cell (or particle)-containing droplets from empty droplets in a ferrofluid flow (Figure 10C). This is because the encapsulation of a single cell or particle causes a significant increase in the droplet size [129]. In addition, Han et al. [134] developed a 3D numerical model to simulate and optimize the magnetic separation of sheath-flow focused diamagnetic particles in ferrofluid flows. Besides the size dependence, negative magnetophoresis has been recently proved by Zhou and Xuan [135] to also be a function of particle shape. They demonstrated a continuous-flow separation of equal-volume spherical- and peanut-shaped PS beads in 0.3× EMG 408 ferrofluid through a T-shaped microchannel. Later, Chen et al. [130] demonstrated in a similar device a morphology-based fractionation of drug-treated yeast cells. They evaluated the separation performance by dividing the cells into the groups of singles, doubles, triples, and others. The authors also developed a 3D numerical model to simulate the cell separation process (Figure 10D). In addition, Zhou et al. [136] achieved a shape-based separation of magnetic particles in diamagnetic fluids using the setup shown in Figure 3B (top). They stated that diamagnetic particles can also be separated by shape in a confined ferrofluid flow because those particles experience similar shape-dependent lateral migration [74] (Figure 3B, bottom).

### 6.2. Two-Sheath-Flow Focusing

**Figure 11:**
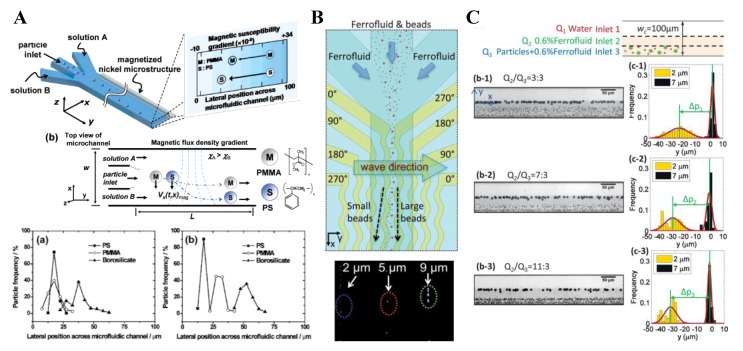
Diamagnetic particle separation in the flow of magnetic fluids with two-sheath-flow focusing. (**A**) Schematic of isomagnetophoresis (top) and measured positions of similar-sized PS, PMMA, and borosilicate (BS) spherical beads (approximately 15 µm in diameter) in conventional magnetophoresis and isomagnetophoresis (bottom). Adapted with permission from Kang et al. [137], © 2007 American Chemical Society. (**B**) Schematic of the separation channel of the ferrofluid-mediated nanocytometry chip (top) and fluorescence microscope snapshot of the separated 2.2, 4.8, and 9.9 µm PS beads in EMG 700 ferrofluid at the channel outlet. Adapted with permission from Kose and Koser [138], © 2012 American Chemical Society. (**C**) Configuration for inlet solutions of the microchannel (top) and the stack images/Gaussian distributions of 2 µm and 7 µm PS beads at the channel outlet. Adapted with permission from Zhou and Wang [95], © 2016 American Institute of Physics.

There are a few studies on the use of two sheath flows for enhanced diamagnetic particle separation in magnetic fluids. Kang et al. [137] developed an improved magnetophoretic method and termed it isomagnetophoresis. This method employs the magnetic susceptibility gradient across a microchannel, which is formed by sandwiching the diamagnetic particle suspension with two sheath flows of high- and low-magnetic susceptibility, respectively (Figure 11A, top). With a permanent magnet and a near-channel nickel microstructure, the authors successfully discriminated similar-sized PS (14.78 µm), poly(methyl methacrylate) (PMMA, 15 µm), and borosilicate (BS, 14.01 µm) beads. The former two particle types cannot be distinguished by conventional magnetophoresis because of their similar diamagnetic susceptibilities (Figure 11A, bottom). Hahn and Park [47] later reported an isomagnetophoretic immunoassay that can detect analytes bound to magnetic nanoparticle labels on diamagnetic microbeads. Kose and Koser [138] presented a low-cost flow-through nanocytometer that utilizes ferrofluid for size-based separation of PS beads. The underlying electrodes carry currents and run parallel to the length of the microchannel, such that ferrofluid-mediated magnetic separation takes place orthogonal to the flow direction (Figure 11B, top). Moreover, the authors used varying electrode gaps to alter locally the balance between magnetic force and torque effects, which makes it possible to sort more than two species at one given excitation frequency. The authors demonstrated in their developed cytometry microchip a rapid separation of 2.2, 4.8, and 9.9 µm PS beads in EMG 700 ferrofluid (Ferrotec) with a high separation efficiency (Figure 11B, bottom). Zhou and Wang [95] demonstrated a novel strategy for separating diamagnetic particles via the multiple fluid interfaces in a three-stream multiphase flow configuration. They injected a suspension of 2 µm and 7 µm PS beads in ferrofluid along with a neighboring bead-free ferrofluid stream and then a water stream (Figure 11C, top). By the end of the straight fluidic channel, the larger beads were focused onto the water–ferrofluid interface while the smaller ones remained near their original entry stream because of the size-sensitive negative magnetophoretic migration. In another study, Munaz et al. [139] used both experiment and simulation to optimize the diamagnetic particle separation in ferrofluids with two or three parallel streams.

### 6.3. Sheath Free

**Figure 12:**
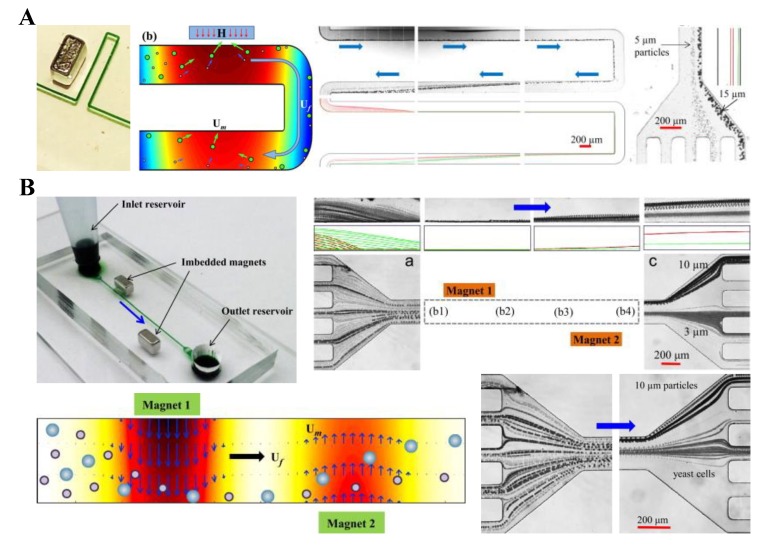
Sheath-free diamagnetic particle separation by size in the flow of magnetic fluids. (**A**) Picture of the microfluidic chip (left) and schematic showing the mechanism of diamagnetic particle separation in the flow of magnetic fluids through a U-shaped microchannel (middle). Adapted with permission from Liang and Xuan [141], © 2012 American Institute of Physics. Comparison of the experimental and numerical images of 5 µm and 15 µm PS beads in 0.5× EMG 408 ferrofluid at different channel locations (right). Adapted with permission from Zhou et al. [142], © 2016 Elsevier B.V. (**B**) Picture of the microfluidic device (upper left) and schematic of the particle separation mechanism (lower left); comparison of the experimental and theoretical images of 3 µm and 10 µm PS beads in 0.05× EMG 408 ferrofluid (upper right); streak images showing the separation of yeast cells and 10 µm PS beads at the inlet and outlet expansions of the microchannel (lower right). Adapted with permission from Zeng et al. [143], © 2013 Elsevier B.V.

As the use of sheath flow(s) not only dilutes the separated particles but also complicates the flow control, several approaches have been proposed to eliminate this component. Kose et al. [140] presented a simple microfluidic platform that used custom-made biocompatible ferrofluids for a label-free separation of both PS beads and live cells. This low-cost platform exploited the differences in the size, shape, and/or elasticity of diamagnetic particles to achieve a rapid and efficient separation. Current-carrying electrodes in quadrature were used to create a periodic magnetic field pattern traveling along the length of the microchannel. The authors demonstrated the platform with the separation of 2.2 µm and 9.9 µm PS beads with 99% efficiency. They also demonstrated shape-based separation of healthy RBCs from sickle cells and bacteria. Liang and Xuan [141] developed a novel microfluidic approach to continuous sheath-free diamagnetic particle separation in the flow of ferrofluids through a U-shaped microchannel (Figure 12A, left). Their approach exploited the repulsive diamagnetic force to pre-focus and separate particles in the two branches of the microchannel (Figure 12A, middle), respectively. The authors demonstrated a continuous-flow separation of 5 µm and 15 µm PS beads in 0.01× EMG 408 ferrofluid. In a later paper from the same group, Zhou et al. [142] developed a 3D numerical model to understand and simulate the diamagnetic particle transport process in the entire microchannel (excluding the outlet). The predicted particle trajectories agree well the experimental observations in a systematic parametric study (Figure 12A, right). In another study, Zeng et al. [143] proposed the use of two offset permanent magnets to separate diamagnetic particles in ferrofluid flow through a straight rectangular microchannel (Figure 12B, upper left). The first magnet, which was placed closer to the microchannel, pre-focused the particle mixture towards the opposing channel sidewall. The second magnet, which was placed farther from the channel than the first magnet, displaced the pre-aligned particles into dissimilar flow paths for a continuous size-based separation (Figure 12B, lower left). The authors demonstrated their system through the separation of 3 μm and 10 μm-diameter PS beads in 0.05× EMG 408 ferrofluid. The experimental results were found to agree well with the predictions of a 3D analytical model (Figure 12B, upper right). The authors also presented a separation of live yeast cells from 10 μm PS beads using their device (Figure 12B, lower right).

**Figure 13:**
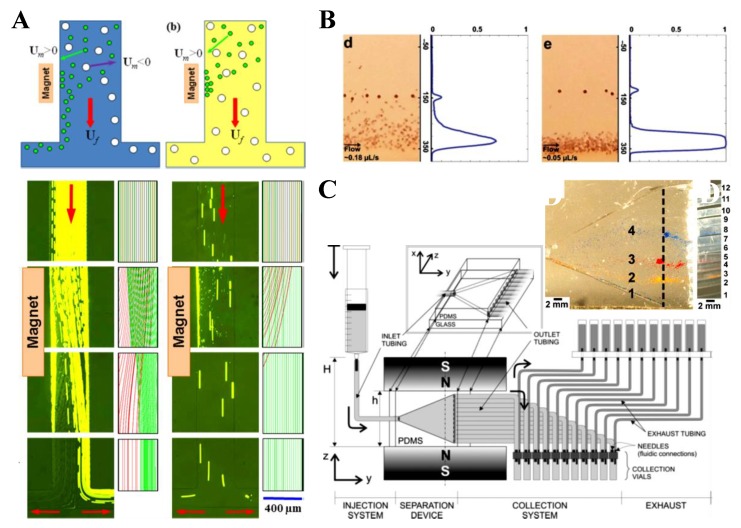
Sheath-free diamagnetic particle separation by non-size properties in the flow of magnetic fluids. (**A**) Schematics comparing the separation of magnetic and diamagnetic particles in ferrofluid (top left) and water (top right); Experimental and theoretical demonstrations of the separation of 2.85 µm magnetic beads and 10 µm PS beads in 0.1× EMG 408 ferrofluid at 240 µL/h (bottom left) and water at 150 µl/h (bottom right). Adapted with permission from Liang et al. [144], © 2013 American Institute of Physics. (**B**) Smartphone images showing the density-based separation of PS beads and blood cells in 50 mM Gadavist solution at the flow rates of 0.18 (left) and 0.05 (right) µL/s. Adapted with permission from Amin et al. [92], © 2016 The Royal Society of Chemistry. (**C**) Schematic representation of the particle separation and collection system (lower) and image of the flow separation of four different Merrifield resins with different amounts of chloromethyl functionality in 250 mM GdCl_3_ solution (upper). Adapted with permission from Winkleman et al. [146], © 2007 American Chemical Society.

There are a few other reports on sheath-free separation of diamagnetic particles in magnetic fluids, where the pre-focusing of particles is actually not needed because of the simultaneous focusing and separation of particles in the flow. Two of these studies are on the separation of particles by magnetic susceptibility. Traditional magnetic separation of particles takes place in the flow of diamagnetic solutions, where magnetic particles are captured leaving diamagnetic particles unaffected by the magnetic field. Liang et al. [144] proposed to replace the diamagnetic solutions with magnetic fluids, such that both magnetic and diamagnetic particles can be manipulated by the magnetic field through positive and negative magnetophoresis, respectively (Figure 13A, top). The authors demonstrated a continuous separation of 2.85 µm magnetic and 10 µm diamagnetic beads in 0.1× EMG 408 ferrofluid through a T-shaped microchannel at the flow rate of 240 µL/h, significantly higher than that of 150 µL/h in water. They also developed a 3D analytical model to simulate the transport and separation of beads in both types of suspending media (Figure 13A, bottom). Later, Zhu et al. [145] used a similar idea to separate mixtures of particles with different magnetic properties in the flow of ferrofluids with custom-defined. They reported the design, modeling, fabrication, and characterization of the separation device. They also used the device to separate magnetic and diamagnetic beads as well as beads with dissimilar magnetizations. Another two studies utilized the magnetic levitation to counterbalance the gravitation for concurrent focusing and separation of diamagnetic particles by density in the flow of paramagnetic solutions. Amin et al. [91] used the setup shown in Figure 4B to demonstrate the separation of PS beads with dissimilar densities and as well PS beads from blood cells in 50 mM Gadavist solution at a flow rate of 180 µL/h (Figure 13B). In an earlier study, Winkleman et al. [146] developed a microfluidic density-based diamagnetic particle separation device based on a similar principle (Figure 13C, lower). They demonstrated the separation of 75–100 µm diameter Merrifield resins that differ in their content of CH_2_Cl groups in an aqueous solution of 250 mM GdCl_3_ over a range of flow rates from 6 to 15 mL/h (Figure 13C, upper). The authors expected their technique to be useful for purifying large quantities of samples because of the continuous flow feature of the demonstrated separation.

### 6.4. Hybrid

**Figure 14:**
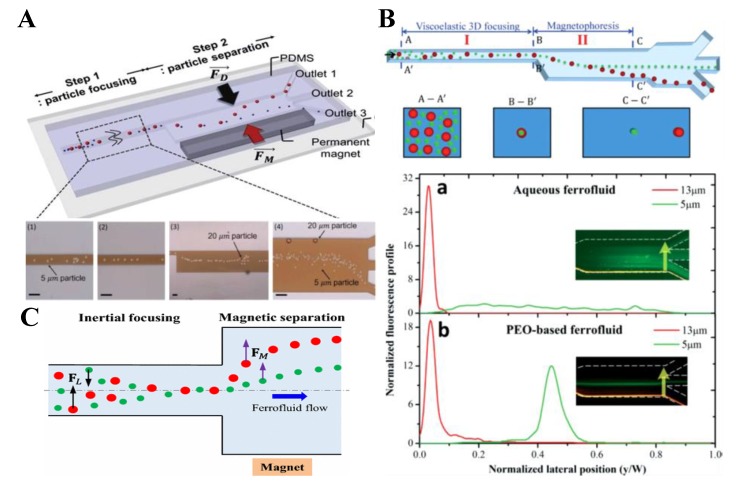
Negative magnetophoresis-based hybrid separation of particles in the flow of magnetic fluids. (**A**) Schematic of the setup (top) and the separation demonstration of elastically focused 5 µm and 20 µm PS beads in the flow of 0.4 wt% PEO and 10 wt% EMG 408 ferrofluid. Adapted with permission from Kim et al. [148], © 2016 The Royal Society of Chemistry. (**B**) Schematic illustration of viscoelastic focusing-enabled diamagnetic particle separation in a PEO-based ferrofluid (top); Comparison of the normalized fluorescence profiles for 5 µm and 13 µm PS beads at the channel outlet in the aqueous ferrofluid and PEO-based ferrofluid, respectively (bottom). Adapted with permission from Zhang et al. [149], © 2016 The Royal Society of Chemistry. (**C**) Schematic illustrating the inertially focused diamagnetic particle separation in a ferrofluid flow. Adapted with permission from Zhou et al. [150], © 2017 Springer.

Owing to the complexity and heterogeneity of biological samples, no single technique is able to separate and sort particles at a high efficiency. It is envisioned that the integration of two or more separation methods, preferably into one microchip, may be a promising field to explore. Recently there has been a significantly growing development in the hybrid techniques that combine active and passive particle separation methods for enhanced sensitivity and flexibility [147]. Among them, the integration of active magnetic separation with passive inertial and/or elastic focusing shows good potentials because of their respective unique features. Kim et al. [148] developed a two-step label-free particle separation technique in the flow of poly(ethylene oxide) (PEO)-based ferrofluid (Figure 14A, top). The first step uses the elastic force in the PEO solution to focus particles into the centerline of the microchannel. The second step uses the diamagnetic repulsion force in the ferrofluid to displace particles towards size-dependent flow paths. The authors demonstrated in their device the separation of 5 µm and 20 µm PS beads (Figure 14A, bottom). They also performed theoretical analysis and numerical simulations to understand the particle transport processes. A similar idea was also employed by Zhang et al. [149] to separate diamagnetic particles in a viscoelastic-based ferrofluid. The authors changed the position of the magnet (Figure 14B, top), which should enhance the separation by reducing the particle traveling distance (and hence the dispersion) in the wide expansion region of the channel. They compared the separation of 5 µm and 13 µm PS beads in between the conventional Newtonian ferrofluid and the viscoelastic ferrofluid (Figure 14B, bottom). In a later study, Zhou et al. [150] exploited the inertial focusing of the ferrofluid flow itself to align diamagnetic particles in a straight rectangular microchannel for a size-based separation (Figure 14C). They also developed a 3D numerical model to simulate the particle focusing and separation processes in the entire microchannel and validated it with the experimental observations at different ferrofluid concentrations and flow rates. In another study, Zhang et al. [151] demonstrated a sheath-free separation of magnetic and diamagnetic particles in the flow of a diluted ferrofluid through a groove-based microchannel. Because of the interaction of positive/negative magnetophoresis with hydrophoresis, 6 µm magnetic beads migrated towards the centerline of the channel while 13 µm diamagnetic beads were directed towards the sidewalls. This separation was found to remain effective in a wide range of flow rates for up to 4.8 mL/h.

### 6.5. Summary

Table 4 summarizes the above-reviewed experimental studies on the continuous-flow separation and sorting of diamagnetic particles and cells (highlighted in bold fonts) using magnetic fluids. We see that the particle flow rate, which is relevant to particle throughput, was smaller than 1 mL/h with just few exceptions. This value is still too low for practical clinic applications that require a flow rate on the order of 10 mL/h or more. One simply way to boost the separation throughput is to use a long and strong magnet to extend the working range of magnetic field as demonstrated by Zhao et al. [133]. Alternatively, the separation throughput can be enhanced through a parallel operation of multiple identical microchannels. 

**Table 4:** Summary of the published papers on diamagnetic particle separation in magnetic fluid flows. Note that the use of biological cells in the separation demonstration has been each highlighted in bold fonts.

Pre-Focused	Particles	Fluid	Magnet		Particle Flow Rate	Reference
			Configuration	Type		
1 sheath	5/10 µm PS	0.79 M MnCl_2_	single	permanent	20–60 µL/h	[90]
1 sheath	5/10 µm PS	0.24–0.48 M MnCl_2_	single	superconducting	70 µL/h	[125]
1 sheath	1/3/6 µm PS	1 M MnCl_2_	single	superconducting	58 µL/h	[126]
1 sheath	8/10 µm PS **RBC/U937**	0–80 mM Gd-DTPA	single	permanent + micromagnet	19.2 µL/h	[127]
1 sheath	1/1.9/3.1/9.9 µm PS	1.2% ferrofluid	single	permanent	180 µL/h	[131]
1 sheath	1/7.3 µm PS **yeast/*E.coli***	1.2% ferrofluid	stacked	permanent	90 µL/h	[132]
1 sheath	5.8/15. µm PS **HeLa RBC**	0.3% ferrofluid	single	permanent	480 µL/h	[128]
1 sheath	cancer cells WBC	0.26% ferrofluid	single	permanent	1.2–6 mL/h	[133]
1 sheath	**cell containing droplets**	0.08% ferrofluid	single	permanent	-	[129]
1 sheath	6 µm spheres/peanuts	0.36% ferrofluid	single	permanent	6 µL/h	[135]
1 sheath	**drug treated yeasts**	0.12% ferrofluid	single	permanent	9 µL/h	[130]
2 sheathes	15 µm PS/PMMA/BS	125 mM Gd-DTPA	single	permanent + micromagnet	-	[137]
2 sheathes	6/10 µm PS+biomarkers	10/25 mM Gd-DTPA	single	permanent + micromagnet	1.2 µL/h	[47]
2 sheathes	2.2/4.8/9.9 µm PS	5.8% ferrofluid	electrodes in quadrature	electromagnet	24 µL/h	[138]
2 sheathes	2/7 µm PS	0.36/0.6% ferrofluid	single	permanent + micromagnet	50–120 µL/h	[95]
2 sheathes	3.2/4.8 µm PS	0.25–1% ferrofluid	array	permanent	60 µL/h	[139]
-	2.2/9.9 µm PS **RBC sickle cells** **bacteria**	customized ferrofluid	electrodes in quadrature	electromagnet	-	[140]
-	5/15 µm PS	0.012% ferrofluid	single	permanent	~20 µL/h	[141]
-	5/15 µm PS	0.6% ferrofluid	single	permanent	450 µL/h	[142]
-	3/10 µm PS **yeast cells**	0.06% ferrofluid	two offset	permanent	10–20 µL/h	[143]
-	10 µm PS 2.85 µm mag	0.12% ferrofluid	single	permanent	240 µL/h	[144]
-	4.2/7.3 µm PS 2.6/7.9 µm mag	1.2% ferrofluid	single	permanent	~200 µL/h	[145]
-	10 µm PS **blood**	50 mM Gadavist	repulsing pair	permanent	~100 µL/h	[91]
-	75–100 µm Merrifield resins	250 mM GdCl_3_	repulsing pair	permanent	6–15 mL/h	[146]
elastic	5/20 µm PS	0.12% ferrofluid	single	permanent	5–200 µL/h	[148]
elastic	5/13 µm PS	0.12% ferrofluid	single	permanent	900 µL/h	[149]
inertial	10/20 µm PS	0.36–0.84% ferrofluid	single	permanent	0.5–1.5 mL/h	[150]
Hydrophoretic	13 µm PS 6 µm mag	0.06% ferrofluid	single	permanent	0.3–4.8 mL/h	[151]

## 7. Particle Medium Exchange

As reviewed above, paramagnetic solutions and ferrofluids have each been widely used for label-free manipulation of diamagnetic particles in microfluidic devices. Their biocompatibility is, however, still a concern. This issue becomes significant if the paramagnetic salt or ferrofluid concentration needs to be high or the exposure time of particles to the magnetic medium needs to be long for high throughput and high volume applications. One way to potentially resolve it is re-suspending particles into a biocompatible buffer immediately after their manipulation in a magnetic fluid. This so-called real time particle washing is one objective of the particle medium exchange step [152]. A number of microfluidic approaches have been developed to achieve on-chip particle medium exchange [153,154,155,156]. Below we first present a short summary on the biocompatibility of magnetic fluids. We then review the published studies on the use of diamagnetic particle deflection across a magnetic fluid for a continuous-flow medium exchange.

### 7.1. Biocompatibility of Magnetic Fluids

Aqueous solutions of simple paramagnet salts such as Mn(II) (e.g., MnCl_2_) and Gd(III) (e.g., GdCl_3_) are not compatible to biological applications [71]. In contrast, aqueous solutions of chelates of Mn(II) (e.g., Mn⋅EDTA) and Gd(III) (e.g., Gd⋅DTPA) become biocompatible [157,158]. Particularly, nine of the Gd(III) chelates (e.g., Gd-DTPA and Gd-dodecane tetraacetic acid (Gd-DOTA) [159]) have been approved by the U.S. Food and Drugs Administration (FDA) for in vivo uses in magnetic resonance imaging (MRI) as contrast agents [160]. However, the concentration of Gd(III) chelates has to be limited, otherwise the viability and growth of the cells exposed to them may be negatively impacted [46,159]. As reviewed above (see Table 1, Table 2, Table 3 and Table 4), commercial ferrofluids (e.g., EMG series water-based ferrofluid like EMG 408 and 700 from Ferrotec) have been increasingly used to manipulate diamagnetic particles. However, their uses in cellular assays are still of significant concerns unless they are sufficiently diluted [93,107,143]. As alternatives, several customized biocompatible ferrofluids have been prepared for the magnetic manipulation of biological cells. They cover the bovine serum albumin (BSA)-coated ferrofluid [161], citrate-stabilized ferrofluid [140], graft copolymer functionalized ferrofluid [128,133,162], and dextran-coated ferrofluid [163]. A good summary of the biocompatibility of magnetic fluids has been provided in the recent review article by Zhao et al. [23] and is hence skipped here.

### 7.2. Single Magnet

**Figure 15:**
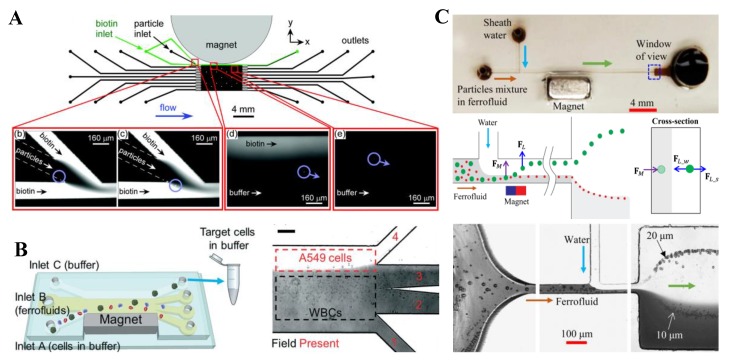
Continuous-flow washing of diamagnetic particles in magnetic fluids. (**A**) Schematic of the device and setup used for the diamagnetic repulsion-based streptavidin-biotin assay (top) and images showing the deflection of a polymer bead across multiple streams (bottom). Adapted with permission from Tarn et al. [164], © 2015 The Royal Society of Chemistry. (**B**) Schematic illustration of a biocompatible cell separation in a multiphase ferrofluid flow (left) and image of the separation/washing process of spiked cancer cells from WBCs at the end of the straight channel section (right). Adapted with permission from Zhao et al. [165], © 2017 The Royal Society of Chemistry. (**C**) Picture of the microfluidic device (top), schematic showing the mechanism of diamagnetic particle separation and washing in an inertial ferrofluid/water co-flow (middle), and experimental demonstration with 20 μm and 10 μm PS beads in 0.75× EMG 408 ferrofluid (bottom). Adapted with permission with Chen et al. [166], © 2017 American Chemical Society.

Tarn et al. [164] demonstrated a proof-of-principle multi-laminar flow assay on the surface of diamagnetic particles (Figure 15A, top). Streptavidin-functionalized polymer beads were suspended in a paramagnetic MnCl_2_ solution that was introduced into a flow-focusing region of the microchip (Figure 15A, bottom). They were deflected through the biotin reagent stream and further into a washing stream by the diamagnetic repulsion, which enabled a one-step multi-laminar flow reaction with detection achieved via fluorescence. Zhao et al. [165] reported a biocompatible label-free cell separation method in the flow of ferrofluids. Their microfluidic device was designed to shorten the exposure time of live cells to the ferrofluid (Figure 15B, left), where the cell sample, ferrofluid, and buffer were injected into the device without pre-mixing. Cells got in contact with the ferrofluid only when they were separated from each other. After separation, larger cancer cells were extracted into the washing buffer. The authors demonstrated the separation of low-concentration cancer cells from WBCs at a 1.2 mL/h throughput with an average 82.2% separation efficiency. In another study, Chen et al. [166] proposed an integration of negative magnetophoresis and inertial focusing for a simultaneous separation and washing of diamagnetic particles from ferrofluid to water (Figure 15C, top). These two operations both took place in the main-branch of the T-shaped microchannel with a permanent magnet placed on one side (Figure 15C, middle). The authors demonstrated the device using a mixture of 10 µm and 20 µm PS beads in 0.75× EMG 408 ferrofluid (Figure 15C, bottom). They demonstrated tunable exiting positions of the beads by varying the flow rate ratio among the two streams or the total flow rate at a fixed flow rate ratio. 

### 7.3. Summary

Table 5 summarizes the above-reviewed studies on the continuous-flow medium exchange for diamagnetic particles suspended in magnetic fluids.

**Table 5:** Summary of the published papers on diamagnetic particle medium exchange in magnetic fluid flows.

Exchange Medium	Particles	Fluid	Magnet		Particle Flow Rate	References
			Configuration	Type		
buffer sheath	4.3 µm PS	0.79 M MnCl_2_	single	permanent	0.52 µL/h	[164]
buffer sheath	5.8/15.7 µm PS cancer cells WBC	0.26% ferrofluid	single	permanent	1.2 mL/h	[165]
water sheath	10/20 µm PS	0.9% ferrofluid	single	permanent	1 mL/h	[166]

## 8. Conclusions and Perspectives

We reviewed the recent advances in label-free manipulations of diamagnetic particles in the confined flow of magnetic fluids. The reported studies have been divided into five primary categories on particle deflection, focusing, enrichment, separation, and medium exchange, respectively. These continuous-flow particle manipulations are each summarized in a corresponding table (Table 1, Table 2, Table 3, Table 4 and Table 5). One can see that most of these manipulations have been demonstrated for PS beads only. This should be mainly due to the standing biocompatibility issue, especially significant if high concentration magnetic fluids must be used to enhance throughput for clinically relevant applications [20,21,22]. While some progress has been made in this direction [23,24], particularly by Mao’s group on the synthesis of customized ferrofluids [128,133], the further development of perhaps even stronger superparamagnetic nanoparticles is definitely beneficial in order to keep the ferrofluid concentration low. Also, the production of commercially available biocompatible ferrofluids is highly desired. Moreover, all reported diamagnetic particle manipulations in0- magnetic fluids have thus far been limited to PS beads or cells with at least a few microns in size. It therefore would be very interesting to see if submicron or even nanoparticles can be manipulated in the flow of magnetic fluids. Yellen’s group [51,52,53,54,55] has made several attempts in this direction with the help of patterned magnetic microstructures though their particle manipulations are free of flows. In addition, there have been limited studies on the use of theoretical or numerical tools to understand the flow of magnetic fluids and the motion of diamagnetic particles suspended therein for a better control of particle transport. This direction becomes relevant and significant when the magnetic force becomes strong enough to affect the magnetic fluid flow [83] or when the magnetic fluid susceptibility becomes strong enough to affect the magnetic field distribution therein [97,166,167]. 

## Figures and Tables

**Table 1:** Summary of the published papers on diamagnetic particle deflection in magnetic fluid flows.

Pre-Focused	Particles	Fluid	Magnet		Particle Flow Rate	References
			Configuration	Type		
-	5/10 µm PS	6/10% MnCl_2_	single	superconducting	400 µL/h	[45]
-	2.2/5/10 µm PS	0.3–1.2% ferrofluid	single	permanent	45–960 µL/h	[79]
-	5/10/15 µm PS	0.04–1 M MnCl_2_	stacked	permanent	3.6–14.4 µL/h	[80]
ferrofluid sheath	4.8/7.3 µm PS	1.2% ferrofluid	single	permanent	300 µL/h	[81]
ferrofluid sheath	3.1/4.8 µm PS	0.1% ferrofluid	single	permanent	60 µL/h	[83]
water sheath	1 µm PS	1.0% ferrofluid	uniform field	electromagnet	500 µL/h	[84]
ferrofluid sheath	7 µm PS ellipsoid	0.6% ferrofluid	Halbach array uniform field	permanent	12 µL/h	[74]
